# The Putatively Specific Synthetic REV-ERB Agonist SR9009 Inhibits IgE- and IL-33-Mediated Mast Cell Activation Independently of the Circadian Clock

**DOI:** 10.3390/ijms20246320

**Published:** 2019-12-14

**Authors:** Kayoko Ishimaru, Shotaro Nakajima, Guannan Yu, Yuki Nakamura, Atsuhito Nakao

**Affiliations:** 1 Department of Immunology, Faculty of Medicine, University of Yamanashi, Yamanashi 409-3821, Japan; ikayoko@yamanashi.ac.jp (K.I.); guannan@yamanashi.ac.jp (G.Y.);; 2 Department of Progressive DOHaD Research, School of Medicine, Fukushima Medical University, Fukushima 960-1247, Japan; 3 Atopy Research Center, Juntendo University School of Medicine, Tokyo 113-8421, Japan

**Keywords:** REV-ERBs, mast cells, IgE, IL-33, circadian clock

## Abstract

The cell-autonomous circadian clock regulates IgE- and IL-33-mediated mast cell activation, both of which are key events in the development of allergic diseases. Accordingly, clock modifiers could be used to treat allergic diseases, as well as many other circadian-related diseases, such as sleep and metabolic disorders. The nuclear receptors REV-ERB-α and -β (REV-ERBs) are crucial components of the circadian clockwork. Efforts to pharmacologically target REV-ERBs using putatively specific synthetic agonists, particularly SR9009, have yielded beneficial effects on sleep and metabolism. Here, we sought to determine whether REV-ERBs are functional in the circadian clockwork in mast cells and, if so, whether SR9009 affects IgE- and IL-33-mediated mast cell activation. Bone marrow-derived mast cells (BMMCs) obtained from wild-type mice expressed REV-ERBs, and SR9009 or other synthetic REV-ERBs agonists affected the mast cell clockwork. SR9009 inhibited IgE- and IL-33-mediated mast cell activation in wild-type BMMCs in association with inhibition of Gab2/PI3K and NF-κB activation. Unexpectedly, these suppressive effects of SR9009 were observed in BMMCs following mutation of the core circadian gene *Clock*. These findings suggest that SR9009 inhibits IgE- and IL-33-mediated mast cell activation independently of the functional circadian clock activity. Thus, SR9009 or other synthetic REV-ERB agonists may have potential for anti-allergic agents.

## 1. Introduction

The circadian clock controls a large proportion of genes in a cyclic manner, thereby regulating the timing of cellular activities [1,2]. The circadian clock consists of a cell-autonomous transcription–translation feedback loop involving several clock genes. Briefly, the transcription factors BMAL1 (*Arntl*) and CLOCK heterodimerize, bind to E-box motifs throughout the genome, and activate transcription of their own repressors *Period (Per1-3)* and *Cryptochrome (Cry1, 2)*. The PER and CRY proteins form oligomers and enter the nucleus, where they inhibit BMAL1/CLOCK activity. This negative-feedback loop takes ~24 h to be completed, with several post-transcriptional regulation. Accordingly, the circadian clock controls periodic expression of thousands of clock-controlled genes (CCGs) with E-box motifs in their promoter/enhancer regions other than Per and Cry.

Previously, we showed that in mouse mast cells, *Clock* binds to E-box motif in the promoter of β-subunit gene of the high-affinity IgE receptor FcεRIβ or IL-33 receptor ST2 in a circadian manner, contributing to day–night variation in IgE- and IL-33-mediated mast cell activation [3,4]. Because IgE- and IL-33-mediated mast cell activation plays a key role in the development and maintenance of allergic diseases [5,6], synthetic compounds capable of modifying the period, phase, or amplitude of clock gene expression in mast cells may have potential as new anti-allergy drugs [7,8].

The nuclear receptors REV-ERB-α (*Nr1d1*) and REV-ERB-β (*Nr1d2*) (REV-ERBs) function as transcriptional repressors. REV-ERBs regulate the expression of genes involved in the control of circadian rhythm, metabolism, and inflammatory response [9,10,11]. As components of the circadian clock, REV-ERBs provide a stabilizing loop that regulates the timing and amplitude of Bmal1 [1,2]. Briefly, the BMAL1/CLOCK heterodimer activates transcription of REV-ERBs and, in turn, REV-ERB-α/REV-ERB-β proteins repress *Bmal1* expression by competing bindings of transcriptional activators, RORα and RORγ, to the ROR-response element (RRE) in the *Bmal1* promoter.

Recent studies have shown that pharmacologically targeting of REV-ERBs using putatively specific synthetic agonists, particularly SR9009 [12], has beneficial effects on circadian rhythm disorders, including jet lag, sleep disturbance, metabolic disease, inflammation, and cancer [12,13,14,15]. For instance, administration of SR9009 induces wakefulness and reduces rapid-eye-movement (REM) and slow-wave sleep in mice [13]. However, it remains unclear whether mast cells express functional REV-ERBs, and if so, whether synthetic REV-ERB agonists such as SR9009 would have beneficial in these cells.

Hence, in this study, we sought to determine whether mast cells express functional REV-ERBs, and if so, whether SR9009 affects IgE- and IL-33-mediated mast cell activation. Our results revealed that REV-ERBs are functional in mast cells, and that SR9009 inhibits IgE- and IL-33-mediated mast cell activation. Unexpectedly, this inhibition was independent of functional clock activity. These findings suggest that SR9009 or other synthetic REV-ERB agonists may have therapeutic potential against allergic diseases.

## 2. Results

### 2.1. Mast Cells Express Functional REV-ERBs

**Figure 1:**
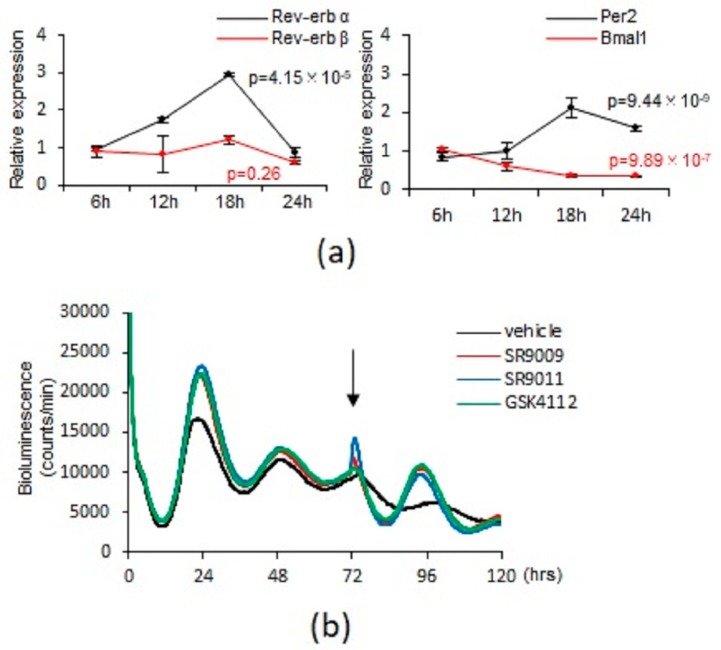
Mast cells express REV-ERBs and synthetic REV-ERB agonists can synchronize the mast cell clockwork. (**a**) Kinetics of the mRNA expression changes of REV-ERB-α, -β, Per2, and Bmal1 at the indicated time points after a medium change in constitutively cultured wild-type BMMCs. The values represent the means ± SD (*n* = 3) (one-way ANOVA). (**b**) Monitoring of PER2^LUC^ bioluminescence of BMMCs derived from PER2^LUC^ knock-in mice after the medium change for 120 h. Synthetic REV-ERB agonists (10 µM) were added to the culture 72 h after the start of the monitoring (black arrow).

First, we investigated whether REV-ERBs are expressed and functional in mast cells. For this purpose, we examined the kinetics of the mRNA levels of REV-ERB-α and REV-ERB-β as well as two other major clock genes, Per2 and Bmal1, in bone marrow-derived mast cells (BMMCs) from wild-type mice. REV-ERB-α and REV-ERB-β mRNAs were expressed at considerable levels comparable to Per2 and Bmal1 in wild-type BMMCs (Threshold Cycle (Ct value) of each gene in the real-time quantitative PCR experiments were as follows; REV-ERB-α: 32~34, REV-ERB-β: 30~32, Per2: 31~33, Bmal1: 30~32). REV-ERB-α, but not REV-ERB-β, mRNA exhibited oscillations (REB-ERB-α: *p* = 4.15 × 10^−5^, REV-ERB-β: *p* = 0.26, one-way ANOVA) with a peak at 18 h following a medium change to synchronize the mast cell clock (Figure 1a). Per2 and Bmal1 mRNA levels exhibited circadian oscillations (Per2: *p* = 9.44 × 10^−9^, Bmal1: *p* = 9.89 × 10^−7^, One-way ANOVA), as previously reported (Figure 1a) [16]. Because no good anti-REV-ERB-α or -β antibody is available, we were unable to confirm REV-ERB-α and -β expression in BMMCs at the protein level. Consistent with a model in which transcription of REV-ERBs is activated by the BMAL1/CLOCK heterodimer [1,2], BMMCs from Clock-mutated mice [17] expressed significantly much lower levels of REV-ERB-α and REV-ERB-β mRNA expression than BMMCs from wild-type mice (Figure S1).

We next examined the effects of SR9009 and other synthetic REV-ERBs agonists SR9011 [12] and GSK4112 [14] on the mast cell clockwork in vitro. We confirmed that treatment of wild-type BMMCs with SR9009, SR9011, or GSK4112 for 24 h at a concentration of 1 or 10 µM did not affect cell viability, as judged by a metabolic assay (NAD(P)H-based: WST-1 assay) (Figure S2a) and expression of Annexin V (Figure S2b); by contrast, a dose of 50 µM exerted cytotoxicity. Therefore, in this study, we used 10 μM, the most commonly used concentration among published studies [12,14,15].

We previously showed that the mast cell clockwork (i.e., the kinetics of PER2 expression) can be evaluated in vitro [3], based on monitoring of bioluminescent emission of BMMCs from Per2^Luc^ knock-in mice, which express PER2 as a luciferase fusion protein [18] (PER2^LUC^ BMMCs). A simple medium change triggers synchronization of the circadian clocks in peripheral cells in vitro [19]. Accordingly, the mast cell clockwork (as reflected by the oscillation of PER2^LUC^) was observed from 0 to 72 h after a media change, as previously described (Figure 1b) [3]. The PER2^LUC^ oscillation may have been limited to 0–72 h due to a lack of oscillator coupling in dissociated cell cultures without internal zeitgebers (‘time givers’ in German), leading to damping of the ensemble rhythm at the population level [3]. We found that addition of 10 μM SR9009, SR9011, or GSK4112 72 h after the medium change recovered the mast cell clockwork (i.e., PER2^LUC^ oscillation) for another 48 h (Figure 1b), suggesting that activation of REV-ERBs by these reagents can synchronize the mast cell clockwork at the population level. Collectively, these results suggested that mast cells express functional REV-ERBs, and that SR9009 or other synthetic agonists can affect the mast cell clockwork.

### 2.2. SR9009 and Other Synthetic Agonists of REV-ERBs Inhibit IgE- and IL-33-Mediated Mast Cell Activation

**Figure 2:**
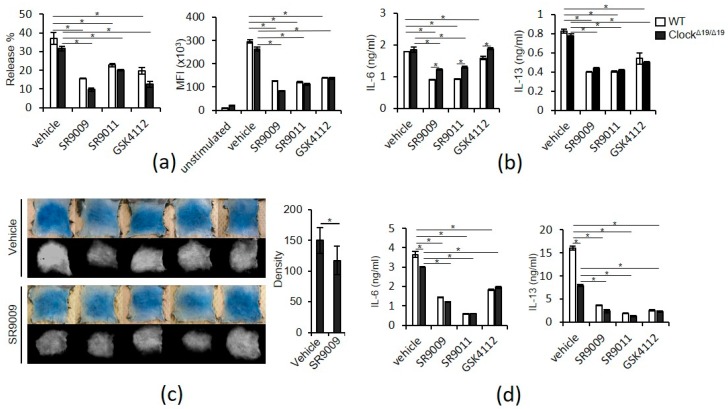
Inhibition of IgE- and IL-33-mediated mast cell activation by synthetic REV-ERB agonists. (**a**) IgE-mediated release of β-hexosaminidase (left) or CD63 upregulation (right) in wild-type and Clock-mutated BMMCs in the presence or absence of 10 μM synthetic REV-ERB agonists (*n* = 3). (**b**) IgE-mediated IL-6 and IL-13 production from wild-type or Clock-mutated BMMCs in the presence or absence of 10 µM synthetic REV-ERB agonists (*n* = 4). (**c**) PCA reactions in wild-type mice i.p. treated with vehicle or 100 mg/kg SR9009. Representative pictures of the skin color reactions (upper left panels) and the digitalized images of the density value evaluations (lower left panels). The quantitative analysis of the data is shown in the right panel. (*n* = 5). (**d**) IL-33-mediated IL-6 and IL-13 production from wild-type or Clock-mutated BMMCs in the presence or absence of 10 µM synthetic REV-ERB agonists (*n* = 4). The values represent the means ± SD (error bars). * *p* < 0.05.

We next examined the effects of SR9009 and other synthetic REV-ERBs agonists on IgE- and IL-33-mediated activation in mast cells. Pretreatment of wild-type BMMCs for 1 h with 10 µM SR9009, SR9011, or GSK4112 inhibited IgE-mediated degranulation, as judged by β-hexosaminidase release, histamine release, and CD63 expression (Figure 2a, Figure S3). IgE-mediated IL-6 and IL-13 protein expression in wild-type BMMCs was also suppressed by pretreatment with 10 µM SR9009, SR9011, and GSK4112 for 1 h (Figure 2b). Importantly, intraperitoneal administration of SR9009 inhibited passive cutaneous anaphylactic (PCA) reaction, a classical in vivo model of IgE/mast cell-mediated skin allergic response, in wild-type mice (Figure 2c). Similarly, pretreatment of wild-type BMMCs for 1 h with 10 µM SR9009, SR9011, or GSK4112 inhibited IL-33-mediated IL-6 and IL-13 protein expression in wild-type BMMCs (Figure 2d). We also found that pretreatment of wild-type BMMCs for 1 h with 10 µM SR9009, SR9011, or GSK4112 inhibited LPS-mediated IL-6 and IL-13 protein expression in wild-type BMMCs (Figure S4a).

Similar to wild-type BMMCs, 10 μM SR9009, SR9011, and GSK4112 inhibited IgE-mediated degranulation and IgE- and IL-33-mediated IL-6 and IL-13 expression in wild-type fetal skin-derived mast cells (FSMCs), mouse connective tissue-type skin-derived mast cells (Figure S5).

Unexpectedly, these suppressive effects of SR9009, SR9011, and GSK4112 were also observed in BMMCs derived from Clock-mutated mice (Figure 2a–d), suggesting that suppression of IgE- and IL-33-mediated mast cell activation by SR9009 or other synthetic REV-ERB agonists does not require functional circadian clock activity. In addition, we found that IL-33-mediated IL-13 production was significantly lower in Clock-mutated BMMCs than in wild-type BMMCs (Figure 2b,d), suggesting that Clock mutation can affect IL-13 production via IL-33 in mast cells.

### 2.3. SR9009 Inhibits IgE- and IL-33-Mediated Activation of the Gab2/PI3K and NF-κB Pathways in Mast Cells

To investigate the mechanisms by which SR9009 suppresses IgE- and IL-33-mediated mast cell activation, we examined the drug’s effects on IgE- and IL-33–induced intracellular signaling pathways leading to degranulation or cytokine expression such as the Gab2/PI3K pathway and NF-κB and p38 MAPK pathways [5,6,20,21].

**Figure 3:**
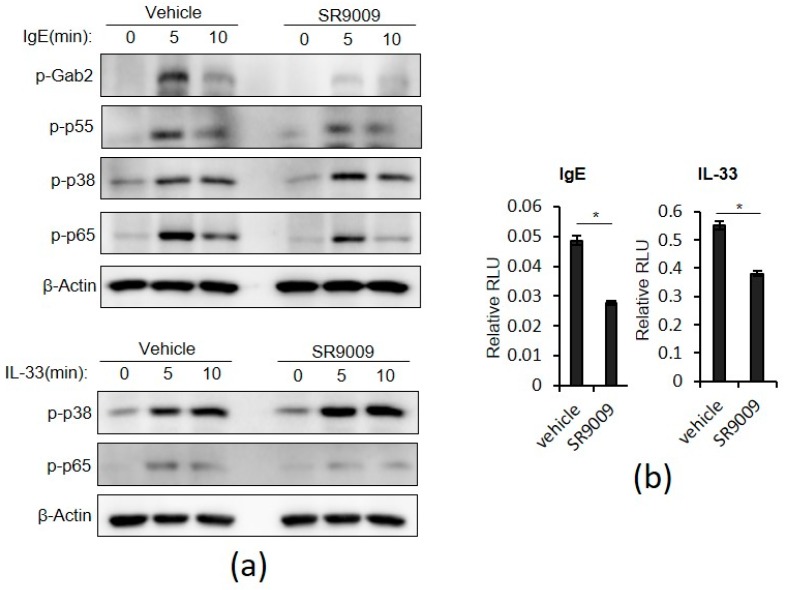
Effects of SR9009 on IgE- and IL-33-mediated intracellular signaling in wild-type BMMCs. (**a**) Western blot analysis of phospho-Gab2, phospho-p55 PI3K, phospho-p38 MAPK, and phospho-p65 in wild-type BMMCs stimulated with IgE or IL-33 for 10 min in the presence or absence of 10 µM SR9009. The level of β-actin is shown at the bottom as a loading control. (**b**) Luciferase assay of NF-κB activity in wild-type BMMCs treated with IgE- or IL-33 in the presence or absence of 10 µM SR9009 (*n* = 3). The values represent the means ± SD. * *p* < 0.05.

The Gab2/PI3K pathway is critical in FcεRI signaling leading to degranulation in mast cells [22]. Briefly, stimulation of FcεRI phosphorylates Gab2 probably by Syk and Src family protein-tyrosine kinases Syk or Lyn. Then, phosphorylated Gab2 binds to the p85 subunit of PI3K and recruits PI3K to its substrate lipids, thereby leading to PLCγ activation and degranulation. Interestingly, SR9009 inhibited IgE-mediated phosphorylation of Gab2 and the p55 subunit of PI3K in wild-type BMMCs (Figure 3a). SR9009 did not affect Gab2 mRNA expression in wild-type BMMCs (Figure S6). In contrast to IgE stimulation, IL-33 did not induce phosphorylaton of Gab2 in wild-type BMMCs.

Both the NF-κB and p38 MAPK pathways mediate IgE- or IL-33-mediated transcriptonal activation of cytokine gene expression [5,6,20,21]. SR9009 inhibited IgE- and IL-33–induced phosphorylation of p65, a subunit of NF-κB, but did not affect IgE- or IL-33–induced phosphorylation of p38 MAPK in wild-type BMMCs (Figure 3a). In addition, a reporter assay showed that SR9009 suppressed IgE- or IL-33-mediated transcriptional activation of NF-κB in BMMCs (Figure 3b). SR9009 also inhibited LPS-mediated transcriptional activation of NF-κB in BMMCs (Figure S4b). Consistent with these findings, SR9009 inhibited IgE-, IL-33-, and LPS-mediated IL-6 and IL-13 mRNA expression in wild-type BMMCs (Figure S7).

We found that SR9009 did not affect surface expression levels of FcεRI or IL-33 receptor ST2 in wild-type BMMCs (Figure S8). Together, these results suggest that inhibition of the Gab2/PI3K and NF-κB pathways, but not p38 MAPK, contributes to the suppressive effects of SR9009 on IgE- or IL-33-mediated degranulation and cytokine gene expression of mast cells.

### 2.4. SR9009 May Inhibit IgE- and IL-33-Mediated Mast Cell Activation Independently of REV-ERBs

The inhibitions of mast cell activation by SR9009 appeared to be independent of the functional circadian clock activity (Figure 2). Thus, we asked whether the inhibitory actions of SR9009 depended on its agonistic function. For this purpose, the effects of SR9009 on IgE- or IL-33-mediated mast cell activation were examined when REV-ERBs expression were knocked-down by specific siRNAs in wild-type BMMCs.

**Figure 4:**
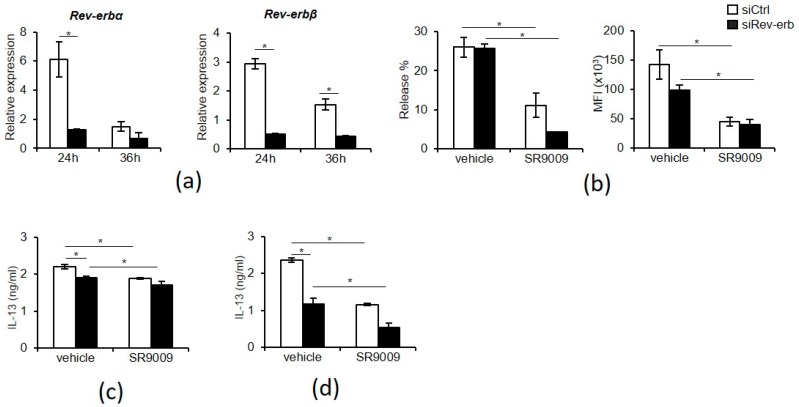
Inhibition of IgE- and IL-33-mediated mast cell activation by SR9009 in REV-ERBs knocked-down BMMCs. (**a**) REV-ERB- α and -β mRNA expression after the specific or control siRNA transfection (*n* = 3). (**b**) IgE-mediated release of β-hexosaminidase (left) or CD63 upregulation (right) in siRNA transfected BMMCs in the presence or absence of 10 μM SR9009 (*n* = 3). (**c**) IgE-mediated IL-13 production from siRNA transfected BMMCs in the presence or absence of 10 µM SR9009 (*n* = 3). (**d**) IL-33-mediated IL-13 production from siRNA transfected BMMCs in the presence or absence of 10 µM SR9009 (*n* = 3). The values represent the means ± SD. *p < 0.05.

Both REV-ERBα and β mRNA expressions were significantly downregulated using the specific siRNAs in wild-type BMMCs by ~80% compared to those in wild-type BMMCs using the control siRNAs (Figure 4a). Unexpectedly, pretreatment of the REV-ERBs knocked-down BMMCs for 1 h with 10 µM SR9009 inhibited IgE-mediated degranulation, as judged by β-hexosaminidase release and CD63 expression (Figure 4b). IgE- and IL-33-mediated IL-13 protein expression was also suppressed by pretreatment with 10 µM SR9009 for 1 h in REV-ERBs knocked-down BMMCs (Figure 4c,d). These results suggest that the inhibitory actions of SR9009 on IgE- and IL-33-mediated mast cell activation may not depend on its agonistic function through REV-ERBs.

## 3. Discussion

Molecular understanding of the circadian clock is opening new therapeutic frontiers for several diseases—including sleep and metabolic disorders, inflammatory diseases, and cancer—through pharmacological targeting of circadian clock components [23]. Given that IgE- and IL-33-mediated mast cell activation is under the control of the circadian clock [3,4] and synthetic REV-ERBs agonists, particularly SR9009, have exhibited many beneficial effects in animal models of circadian-related disorders [12,13,14,15], this study sought to determine whether mast cells express functional REV-ERBs and SR9009 affects IgE- and IL-33-mediated mast cell activation. The current results suggest that SR9009 or other synthetic REV-ERB agonists can synchronize the mast cell clockworks and inhibit IgE- and IL-33-mediated mast cell activation.

SR9009 significantly inhibited IgE- and IL-33-mediated mast cell activation (Figure 2). The Gab2/PI3K pathway is critical in FcεRI signaling leading to degranulation in mast cells [22] and the NF-κB pathway mediates IgE- or IL-33-mediated transcriptonal activation of cytokine gene expression [5,6,20,21]. Thus, it is likely that SR9009 and other synthetic REV-ERB agonists inhibited IgE-mediated degranulation and IgE- and IL-33-mediated IL-6 and IL-13 expression through the suppression of the Gab2/PI3K and NF-kB pathways, respectively. Because the Gab2/PI3K pathway play a partial role in IgE-mediated cytokein expression [22], inhibition of this pathway by SR9009 may be also involved in the suppression of IgE-mediated IL-6 and IL-13 expression.

It remains to be determined how SR9009 inhibits the Gab2/PI3K and NF-κB pathways. However, regarding the NF-κB pathway, there were several reports that address the inhibitory mechanisms of SR9009 on the NF-κB pathway, including transcriptional repression of NF-κB–related genes such as IL-6 [24], p65 [25], and induction of FABP4, an intracellular lipid-binding protein [26]. In contrast, it remains totally unclear how SR9009 inhibits IgE-mediated Gab2/PI3K activation in mast cells.

Pretreatment of Clock-mutated BMMCs with SR9009 and other synthetic REV-ERBs agonists for 1 h can suppress IgE- and IL-33-mediated activation, as in wild-type BMMCs (Figure 2). Moreover, given that treatment of wild-type PER2*^LUC^* BMMCs with SR9009, SR9011, or GSK4112 did not affect PER2*^LUC^* expression 1 h after the addition of the agent (Figure 1b), the suppressive effects of SR9009 or other synthetic REV-ERB agonists may be independent of functional circadian clock activity and PER2 expression in mast cells. The observation that treatment of wild-type BMMCs with SR9009 for 1 h did not affect the expression of FcεRI or ST2 (Figure S8) also support this idea, as expression of FcεRI and ST2 is under the control of mast cell clock activity [3,4].

Surprisingly, SR9009 inhibited IgE- or IL-33-mediated activation in REV-ERBs knocked-down BMMCs (Figure 4). Thus, the inhibitory actions of SR9009 on IgE- and IL-33-mediated mast cell activation may be independent on its agonistic function through REV-ERBs. Most recently, Dierickx et al. reported that SR9009 has REV-ERB-independent effects on cell proliferation and metabolism [27]. They showed that SR9009 can decrease cell viability, rewire cellular metabolism, and alter gene transcription in hepatocytes and embryonic stem cells derived from REV-ERB-α and -β double knockout mice although the mechanisms remain unclear. Thus, SR9009 and possibly other SR9009-related synthetic REV-ERB agonists might inhibit IgE- and IL-33-mediated mast cell activation independently of REV-ERBs. However, it should be noted that REV-ERBs knocked-down BMMCs still express REV-ERB-α and REV-ERB-β mRNAs, albeit at very low levels and SR9009 could exert its function through the residual expression of REV-ERBs.

Clock mutation decreases IL-33-mediated IL-13 production in BMMCs (Figure 2b,d). Kawauchi et al. reported that IL-33-mediated IL-6 and IL-13 production exhibit a time-of-day–dependent variation in synchronized, but not Clock-mutated, BMMCs [4]. Thus, it is likely that Clock may be involved in the circadian regulation of IL-33-mediated IL-13 production, although the mechanisms involved remain to be determined. 

In summary, our findings show that activation of REV-ERBs by SR9009 or other synthetic REV-ERBs agonists can inhibit IgE- and IL-33-mediated activation of mast cells in association with suppression of the Gab2/PI3K and NF-κB pathways. Thus, modulation of REV-ERB activity by synthetic REV-ERB agonists may have potential for broad ranges of allergic diseases.

## 4. Materials and Methods

### 4.1. Materials

Reagents used in this study were acquired from the indicated suppliers: SR9009 (Merck Millipore, Burlington, MA, USA); SR9011, GSK4112, LPS, and Evans blue (Sigma-Aldrich, St. Louis, MO, USA); recombinant mouse IL-3 (PeproTech, Rocky Hill, NJ, USA); recombinant mouse IL-33 (R & D Systems, Minneapolis, MN, USA); anti-TNP IgE, anti-DNP mouse IgE mAb, anti-mouse CD16/32, PE-conjugated anti-mouse c-kit Ab, and APC-conjugated anti-mouse ST2 Ab (BD Bioscience, San Jose, CA, USA); DNP-BSA (Cosmo Bio, Tokyo, Japan); APC-conjugated anti-mouse CD63 Ab, FITC-conjugated anti-mouse FcεRIα (BioLegend, San Diego, CA, USA); anti-phospho-NF-κB p65 Ab (Ser536; #3033), anti-phospho-p38 MAPK Ab (Thr180/Thy182; #4511), anti-phospho-Gab2 Ab (Tyr452; #3882), anti-phosph-PI3 Kinase p85 (Tyr458)/p55 (Tur199) Ab (#4228), and anti-β-actin Ab (#4970) (Cell Signaling Technology, Danvers, MA, USA).

### 4.2. Mice

Male 6–8-week-old C57BL/6 mice (Japan SLC, Tokyo, Japan), Per2*^Luciferase^* (Per2*^Luc^*) knock-in mice (C57BL/6 background) where PERIOD2 (PER2) is expressed as a luciferase fusion protein [18], and C57BL/6 Clock*^Δ19/Δ19^* mice [17] were kept under 12-hour light / 12-hour dark conditions. Clock*^Δ^^19/Δ19^* mice have an A-to-T point mutation in the 5’ splice site of intron 19 and consequently an in-frame deletion of the whole exon 19 (Clock*^Δ19/Δ19^*), resulting in the loss of normal transcriptional activity [17]. All animal experiments were approved by the Institutional Review Board (IRB) of the University of Yamanashi and carried out according to IRB guidelines (ethics committee: Koji Moriishi, Toshiyuki Oda, Hiroaki Nagatomo, Hiroyuki Narita, Jiang Ling, Junichi Miyazaki, Kazuhiro Mori, and Teruhiko Wakayama, approval code: A28-36, 14 August 2019).

### 4.3. Preparation of Bone Marrow-Derived Mast Cells (BMMCs) and Fetal Skin-Derived Mast Cells (FSMCs)

BMMCs were prepared from femoral bone marrow cell suspensions from male C57BL/6 mice, Per2*^Luc^* mice, or Clock*^Δ19/Δ19^* mice as previously described [3]. Briefly, crude bone marrow cells were cultured in RPMI 1640 supplemented with 10% fetal bovine serum, 2 mM L-glutamine, 10 mM nonessential amino acids, penicillin–streptomycin, 10 mM sodium pyruvate, and 50 μM 2-ME (complete RPMI 1640) in the presence of 10 ng/mL recombinant mouse IL-3 (rmIL-3). Floating cells were refreshed twice per week, and further expanded for 2–4 weeks in fresh complete RPMI 1640 supplied with rmIL-3. Finally, the cells (>90% FcεR1^+^c-kit^+^) were used as BMMCs without further purification.

Fetal skin mast cells (FSMCs) were generated from fetal skin of C57BL/6 mice on day 14, as described previously [28]. Briefly, fetal skin was treated with trypsin diluted in medium to form a single cell suspension. Then the cells were cultured in complete medium containing 20 ng/mL rmIL-3 and 20 ng/mL recombinant mouse SCF. After 2 weeks, non-adherent and loosely-adherent cells were collected, and mast cells were collected by Percoll density-gradient centrifugation. Finally, the cells (>90% FcεR1^+^c-kit^+^) were used as FSMCs.

### 4.4. Quantitative Real-Time PCR

Total RNA was isolated from BMMCs using the RNeasy Mini Kit (QIAGEN, Valencia, CA, USA). RNA was quantified on a NanoDrop ND-1000 spectrophotometer (Thermo Fisher Scientific, Waltham, MA, USA). Complementary DNA (cDNA) was synthesized using the ReverTra Ace RT-PCR Kit (TOYOBO, Osaka, Japan). Quantitative real-time PCR (qPCR) was performed on a Step One Plus Fast Real-Time PCR System (Applied Biosystems, Carlsbad, CA, USA) using qPCR Master Mix (Applied Biosystems) with specific primers and probes against mouse *REV-ERB-α*, *IL-13* (Applied Biosystems), *REV-ERB-β*, *IL-6*, and *GAPDH* (IDT; Coralville, IA, USA). qPCR data were normalized against the corresponding levels of *GAPDH* mRNA.

### 4.5. Measurement of Bioluminescence in BMMCs Generated from Per2^Luc^ Mice

BMMCs generated from Per2*^Luc^* knock-in mice were centrifuged at 1500 rpm for 5 min, placed in a 35 mm Petri dish and incubated at 37 °C. As previously described, bioluminescence was monitored for 120 h at 10-min intervals using a dish-type luminometer (Kronos DioAB-2550; ATTO, Tokyo, Japan) [3]. After 72 h of medium change for synchronization, 10 μM REV-ERB agonists were added to the culture.

### 4.6. Flow Cytometry Analysis

BMMCs were stained with PE-conjugated anti-mouse c-kit, FITC-conjugated anti-mouse FcεRI, APC-conjugated anti-mouse ST2, or APC-conjugated anti-mouse CD63 in the presence of rat anti-mouse CD16/32. After washing with PBS, the stained cells were analyzed on a BD Accuri C6 flow cytometer (BD Biosciences). Flow cytometry data were analyzed using the CellQuest software (BD Biosciences).

### 4.7. Stimulations of BMMCs or FSMCs

BMMCs or FSMCs were incubated with 1 µg/mL anti-DNP mouse IgE mAb for 1 h at 4 °C and, then, incubated with or without REV-ERB agonists for 1 h at 37 °C. Then, these cells were stimulated with 1 µg/mL of anti-mouse IgE antibody for 1 h at 37 °C (for β-Hexosaminidase or histamine measurement) or for 6 h at 37 °C (for IL-6 or IL-13 measurement). BMMCs or FSMCs were incubated with or without REV-ERB agonists for 1 h at 37 °C and then stimulated with 1 ng/mL IL-33 or 1 µg/mL LPS for 6 h at 37 °C, followed by measurement of IL-6 and IL-13 in the culture supernatants.

### 4.8. Enzyme-Linked Immunosorbent Assay (ELISA)

Concentrations of IL-6, IL-13, and histamine in supernatants of cell cultures were determined by ELISA. Kits for mouse IL-6 (R & D Systems), mouse histamine (Oxford Biomedical Research, Rochester Hills, MI, USA), mouse IL-13 (eBioscience/Thermo Fisher Scientific) were obtained from the indicated suppliers.

### 4.9. β-Hexosaminidase Release Assay

BMMCs or FSMCs were incubated with 1 µg/mL anti-DNP mouse IgE mAb for 1 h at 4 °C, and then stimulated with 1 µg/mL of anti-mouse IgE antibody with or without REV-ERB agonists for 1 h at 37 °C. Total release was obtained by adding 1% Triton buffer for 40 min. Supernatants were collected from each well and mixed with *p*-nitrophenyl-*N*-acetyl-β-d-glucosaminide to determine the enzymatic activity of the released β-hexosaminidase. After 90 min at 37 °C, the reaction was stopped by addition of 0.2 M glycine solution, and OD (405 nm) was measured on a spectrophotometer.

### 4.10. Western Blot Analysis

Ten minutes after various stimulations, BMMCs were lysed in RIPA buffer (25 mM Tris-HCl, pH 7.6, 150 mM NaCl, 1% Triton ×100, 1% sodium deoxycholate, 0.1% SDS) with protease inhibitor cocktail (Merck Millipore, Burlington, MA, USA) and vanadate (FUJIFILM Wako Pure Chemical Corporation, Osaka, Japan). Cell lysate was dissolved in sample buffer containing 50 mM dithiothreitol and bromophenol blue, and then boiled for 5 min. Protein concentrations were measured on a NanoDrop ND-1000. Proteins were subjected to SDS-PAGE and transferred to polyvinylidene fluoride membranes. Blots were immersed in 5% milk blocking solution for 1 h at room temperature (RT), followed by incubation with primary antibody solution overnight at 4 °C. Membranes were washed three times with TBS/T, and then incubated in a secondary antibody solution for 1 h at RT. Immunoreactive proteins were visualized using ECL Prime (GE Healthcare).

### 4.11. Reporter Assays

Using the Mouse Macrophage Nucleofector kit (VPA-1009; Lonza, Basel, Switzerland), BMMCs were transiently transfected with the pNFκB-Luc reporter plasmid, with pRL-CMV as an internal control. After 24 h, the transfected BMMCs were stimulated with IL-33 (1 ng/mL) or anti-mouse IgE antibody (1 μg/mL) or LPS (1 μg/mL) in the absence or presence of REV-ERBs agonists. Relative NF-κB luciferase activity was normalized against transfection efficiency.

### 4.12. Cell Viability Assay

After stimulations with synthetic REV-ERB agonists for 24 h, cell viability was monitored using the Cell Counting Kit-8 (Dojindo Laboratory, Kumamoto, Japan) and PE-Annexin V apoptosis detection kit (BD Bioscience, San Jose, CA, USA).

### 4.13. Passive Cutaneous Anaphylaxis

Mouse anti-TNP IgE (100 ng) was intradermally injected into dorsal skin. After 24 h, 15% Cremophor (vehicle) or SR9009 (100 mg/kg) was intraperitoneally injected. After 1 h, the mice were challenged by intravenous injection with 2.5mg/kg of DNP-BSA in PBS containing 0.2% Evans blue dye. Vascular permeability was visualized 40 min later as blue staining of the injection areas on the inside of the skin. Staining sites were digitized using a high-resolution color camera, and images were saved JPEG files. The images analyzed using ImageJ 1.43 (NIH, Bethesda, MD, USA) as previously described [3]. Briefly, the color-scale images (the upper panels) were converted to HSB (hue/saturation/brightness) stack images, which were then split into hue, saturation, and brightness images. The blue-stained areas were selected from the hue image using the threshold tool, afterwards these images were combined with the saturation image. The density values for the blue-stained areas were measured using the analyze tool.

### 4.14. siRNAs Experiments

All siRNAs were purchased from Invitrogen (now Thermo Fisher, Waltham, MA, USA). Specific siRNAs against NR1D1 (stealth^TM^ RNAi; Nr1d1MSS211361 [3_RNAI]) or NR1D2 (stealth^TM^ RNAi: Nr1d2MSS221355 [3_RNAI]) were used.

Transfection of wild-type BMMCs were performed with Mouse Macrophage Nucleofector Kit (VPA-1009; Lonza, Basel, Switzerland). BMMCs were suspended in Nucleofector Solution to a final concentration of 2 × 10^6^ cells/100μL. The cells were transfected with 500 nM negative control or 250 nM specific siRNA of NR1D1 and NR1D2, respectively, using the Nucleofector II (Amaxa Biosystems, now Lonza) program Y-001. Subsequently, the transferred cells were placed in a 20% FBS medium and cultured in a 24-well plate. After 24 h, the transfected BMMCs were stimulated with anti-mouse IgE antibody (1μg/mL), IL-33 (1 ng/mL), or LPS (1 μg/mL) in the absence or presence of 10 μM SR9009.

### 4.15. Statistical Analysis

Statistical analyses were performed using the unpaired Student’s *t*-test for two-group comparisons. Multigroup comparisons were analyzed by one-way ANOVA with Tukey–Kramer post hoc test. A value of *p* < 0.05 was considered to be significant.

## Supplementary Materials

Supplementary materials can be found at https://www.mdpi.com/1422-0067/20/24/6320/s1.

## Abbreviations

MAPK	Mitogen-activated protein kinase
NF-κB	Nuclear factor κB
