# Supplementary material for: The Putatively Specific Synthetic REV-ERB Agonist SR9009 Inhibits IgE- and IL-33-Mediated Mast Cell Activation Independently of the Circadian Clock
Source: Int J Mol Sci. 2019 Dec 14;20(24):6320. doi: 10.3390/ijms20246320 (PMC6941044; doi:10.3390/ijms20246320)
Supplement: Supplementary file 1 [file ijms-20-06320-s001.pdf]

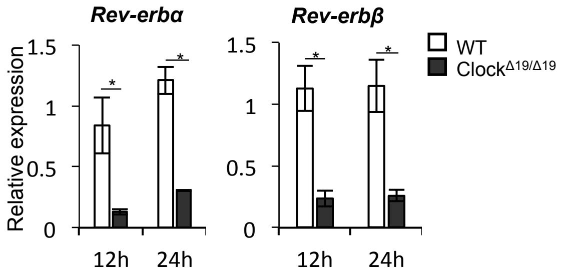

Figure S1. Severe reduction of REV-ERB-α, and –β mRNA expression in Clock-mutated BMMCs. Kinetics of REV-ERB-α, and - β mRNA expression levels in wild-type and Clock-mutated BMMCs are shown. BMMCs were consistently cultured in vitro after a medium change and then mRNA was extracted, and a qPCR analysis was performed for REV-ERB-α and - β mRNA at the indicated time points. The values represent the means ± SD. *$P<0.05$ ($n = 3$).

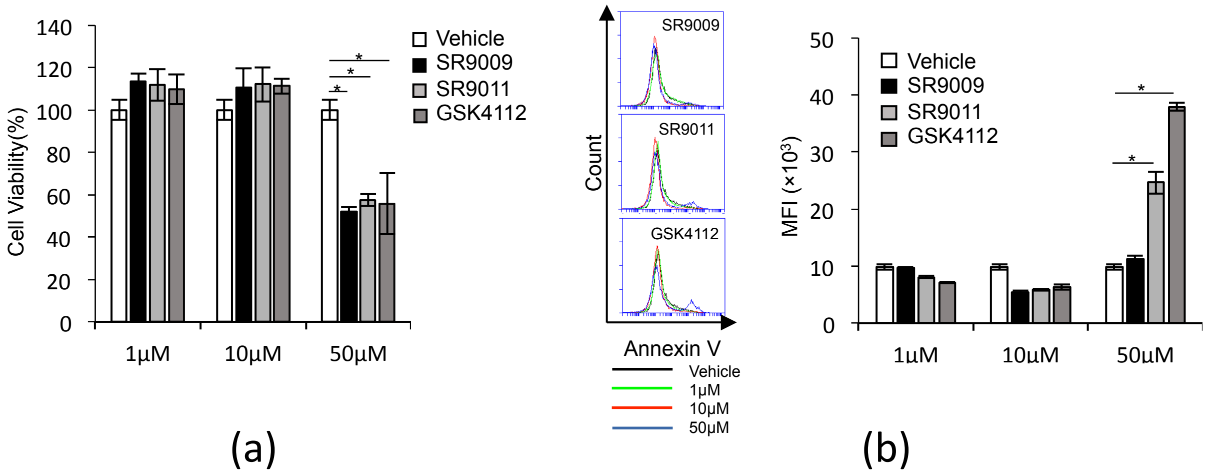

Figure S2. The effects of synthetic REV-ERB agonists on mast cell viability. Wild-type BMMCs were cultured in the presence or absence of the indicated doses of synthetic REV-ERB agonists and their cytotoxicity was quantified via a metabolic assay [NAD(P)H-based: WST-1] (left) and Annexin V staining using flow cytometry (right). The values represent the means ± SD. *P<0.05 (n = 3)

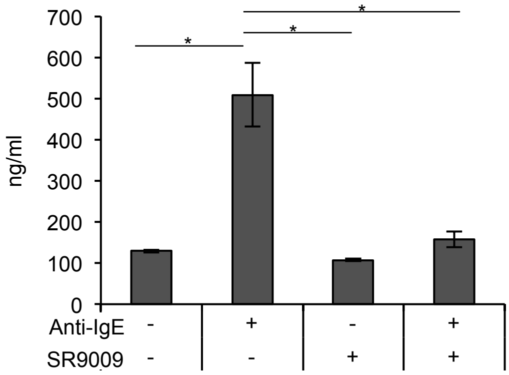

Figure S3. SR9009 inhibits IgE-mediated histamine release from wild-type BMMCs. IgE-mediated release of histamine from wild-type BMMCs treated with or without 10 μM SR9009 were measured using histamine ELISA. The values represent the means ± SD. *P<0.05 (n = 3)

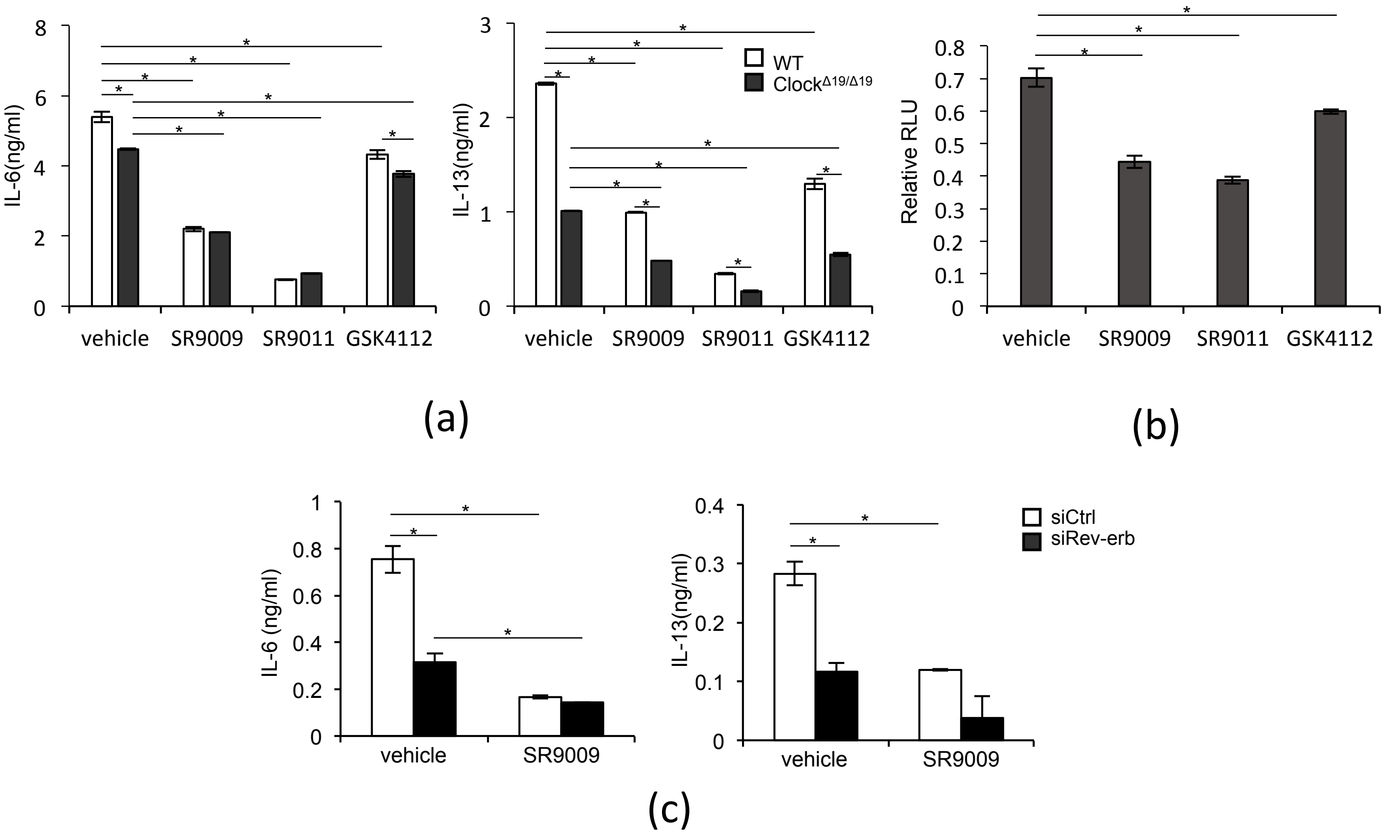

Figure S4. Inhibition of LPS-mediated mast cell activation by synthetic REV-ERB agonists. (a) LPS-mediated IL-6 and IL-13 production from wild-type or Clock-mutated BMMCs in the presence or absence of 10 μM synthetic REV-ERB agonists (n = 4). (b) Luciferase assay of NF-κB activity in wild-type BMMCs treated with LPS in the presence or absence of 10 μM SR9009 (n = 3). (c) LPS-mediated IL-6 and IL-13 production from control or Rev-erbs double-knock down BMMCs in the presence or absence of 10 μM SR9009. The values represent the means ± SD. *P<0.05

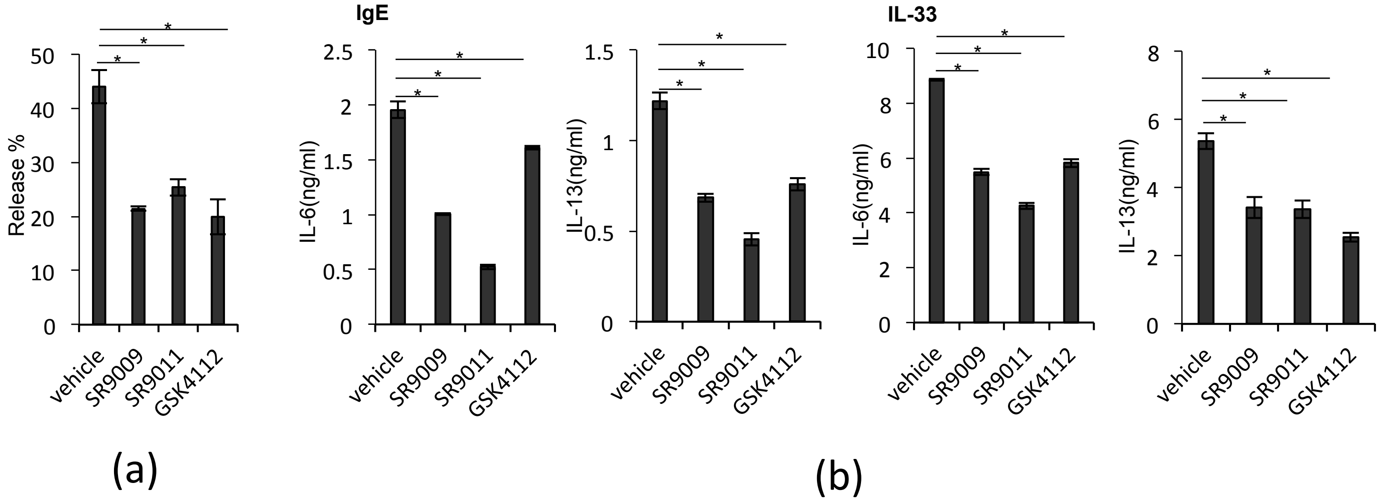

Figure S5. Inhibition of IgE- and IL-33-mediated degranulation or IL-6/IL-13 production by synthetic REV-ERB agonists in fetal skin-derived mast cells (FSMCs). (a) IgE-mediated release of β-hexosaminidase in wild-type FSMCs in the presence or absence of 10 μM synthetic REV-ERB agonists (n = 3). (b) IgE-or IL-33-mediated IL-6 and IL-13 production from wild-type FSMCs in the presence or absence of 10 μM synthetic REV-ERB agonists (n = 3). The values represent the means ± SD. *P<0.05

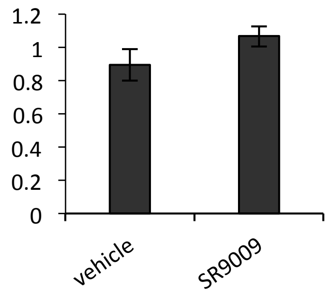

Figure S6. Gab2 mRNA expression was not affected by SR9009. Gab2 mRNA expression was analyzed after 2 hours treated vehicle or 10μM SR9009. (n=3)

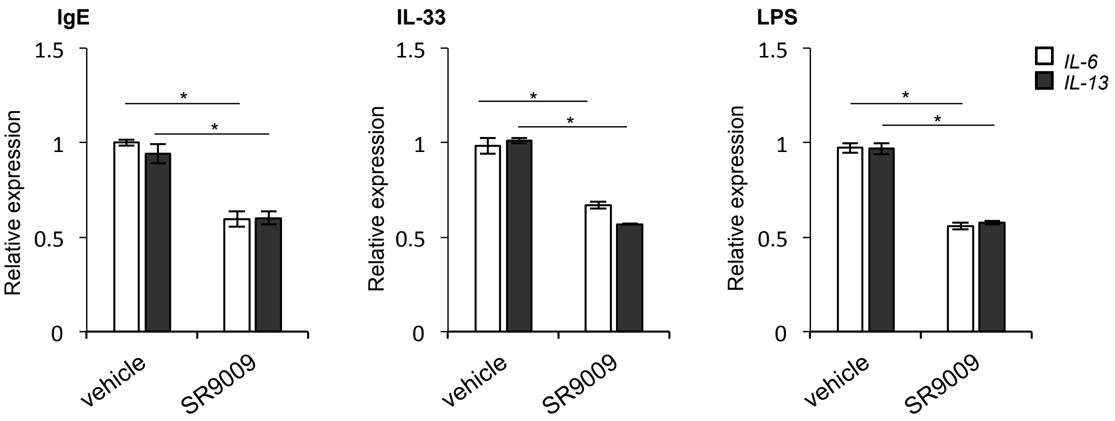

Figure S7. Inhibition of IgE-, IL-33- and LPS-mediated IL-6/IL-13 mRNA expression by SR9009. IgE-, IL-33- or LPS-mediated IL-6 and IL-13 mRNA expression from wild-type BMMCs in the presence or absence of 10 μM SR9009(n = 3). The values represent the means ± SD. *P<0.05 (n=3)

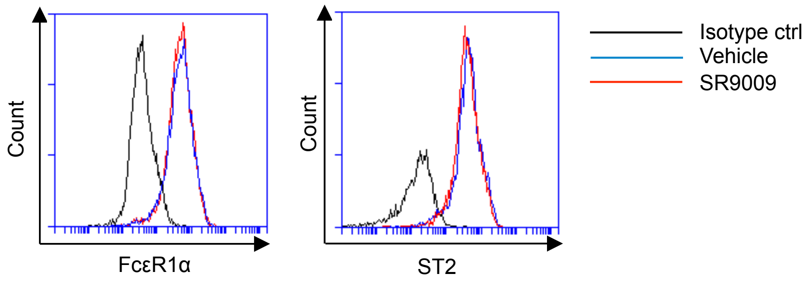

Figure S8. Comparable expression of FcεRI and ST2 in wild-type BMMCs treated with or without SR9009. A quantitative analysis of FcεRIα(left) and ST2(right) levels in wild-type BMMCs treated with or without 10 μM SR9009 for 1 hour by flow cytometory (n = 3), and representative histograms were shown.
